# Morphokinetic behavior in embryos leading to biochemical pregnancy

**DOI:** 10.5935/1518-0557.20250010

**Published:** 2025

**Authors:** Amanda Setti, Daniela Braga, Patricia Guilherme, Assumpto Iaconelli Jr., Edson Borges Jr.

**Affiliations:** 1 Sapientiae Institute - Centro de Estudos e Pesquisa em Reprodução Humana Assistida, São Paulo, SP, Brazil; 2 Fertility Medical Group/FertGroup Medicina Reprodutiva São Paulo, SP, Brazil

**Keywords:** biochemical pregnancy, embryo development, embryo morphokinetics, intracytoplasmic sperm injection

## Abstract

**Objective:**

It has been observed that faster developmental kinetics are linked with a higher number of embryo cells, improved blastocyst development, and increased rates of implantation and pregnancy. However, the embryonic morphokinetics’ predictive value for biochemical pregnancy (BP) outcomes has been minimally investigated. The objective of this study was to investigate whether embryos leading to BP behave morphokinetically differently than those leading to positive or negative pregnancy result.

**Methods:**

This case-control study performed in a private university-affiliated IVF center, included 1248 transferred embryos from 753 women undergoing ICSI cycles between March 2019 and April 2022. Patients were split into three groups according to the pregnancy outcome: Biochemical Group (n=30 cycles/54 transferred embryos), consisting of patients with a BP; Positive Group (n=255 cycles/444 transferred embryos), consisting of patients with a positive pregnancy result (clinical pregnancy); and Negative Group (n=468 cycles/750 transferred embryos), consisting of patients with a negative pregnancy result. Kinetic markers from the point of insemination were recorded in the EmbryoScope incubator.

**Results:**

Embryos resulting in BP behaved similarly to those embryos resulting in a clinical pregnancy. Embryos resulting in a negative pregnancy showed significantly slower embryo development and KIDScore ranking compared to both Biochemical and Positive groups.

**Conclusions:**

Embryos that resulted in a BP did not display evidence of abnormal morphokinetics on time-lapse imaging. Further research is needed to identify factors that can predict and prevent biochemical pregnancy.

## INTRODUCTION

Human reproduction is characterized by its low efficiency. It’s estimated that a staggering 70% of all pregnancies do not result in live births, with 25% to 50% of these resulting in what are known as biochemical pregnancies (BP) (Larsen *et al.*, 2013). A BP is a very early pregnancy loss, where the initial serum or urine beta human chorionic gonadotropin (β-hCG) pregnancy test returns a positive result, but an embryonic sac with a fetal heartbeat is undetected via ultrasound at 6-7 weeks of gestation, thus the pregnancy does not develop into a clinical pregnancy (Annan *et al.*, 2013).

In most cases, BP losses remain undetected in natural pregnancies due to the lack of significant changes in the menstrual cycle. However, for patients who are undergoing treatments involving assisted reproduction technology (ART), where there is active monitoring of β-hCG levels following embryo transfer, BPs are identified in as many as 20% of the cycles (Zeadna *et al.*, 2015).

A positive β-hCG test indeed confirms that at least one embryo has progressed to the advanced preimplantation stage and tried to implant (Annan *et al.*, 2013). Traditionally, a positive test, compared to a previously negative β-hCG test, has been considered a more reliable predictor of a successful pregnancy in subsequent ART cycles (Levy *et al.*, 1991a, 1991b; Bates & Ginsburg, 2002; Haas *et al.*, 2012). However, recent research indicates that recurrent BP can lead to less favorable ART results, and a correlation has been observed between the number of losses and unsuccessful outcomes (Kolte *et al.*, 2014; Maesawa *et al.*, 2015; Yang *et al.*, 2015).

Even though BP occur frequently, the exact causes and contributing factors are still not clearly understood (Lee *et al.*, 2017). Very early pregnancy loss is believed to be influenced not only by endometrial receptivity but also by embryo quality (Salumets *et al.*, 2006; Shapiro *et al.*, 2011; Yang *et al.*, 2015; Lee *et al.*, 2017). Time-lapse imaging (TLI) has been employed to evaluate a range of morphokinetic parameters from the point of insemination to blastulation, and their correlation with both laboratory and clinical outcomes. It has been observed that faster developmental kinetics are linked with a higher number of embryo cells (Coticchio *et al.*, 2018), improved blastocyst development (Dal Canto *et al.*, 2012; Kirkegaard *et al.*, 2013), and increased rates of implantation (Meseguer *et al.*, 2011; Basile *et al.*, 2015) and pregnancy (Lemmen *et al.*, 2008; Meseguer *et al.*, 2012). However, the embryonic morphokinetics’ predictive value for biochemical pregnancy outcomes has been minimally investigated. Therefore, the aim of this study was to investigate whether embryos leading to biochemical pregnancy behave morphokinetically differently than those leading to positive or negative pregnancy result.

## MATERIAL AND METHODS

### Patients and Experimental design

**Figure 1:**
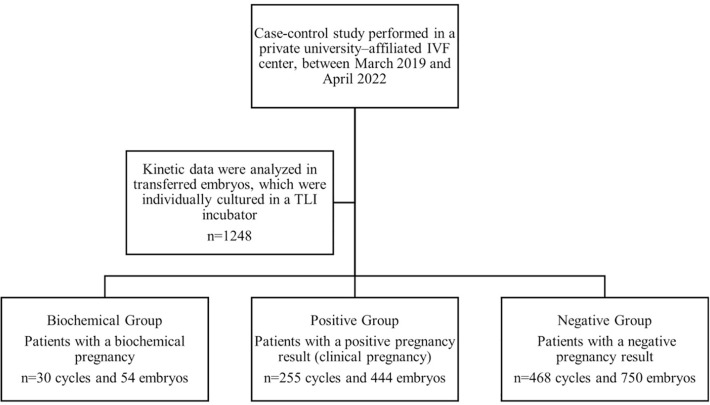
Study design.

This case-control study was performed in a private university-affiliated IVF center, between March 2019 and April 2022. Kinetic data were analyzed in 1248 transferred embryos, which were individually cultured in a TLI incubator (EmbryoScope+, Unisense Fertilitech, Aarhus, Denmark) until day five of development, derived from 753 patients undergoing ICSI cycles. Timing of specific events from the point of insemination was determined using time-lapse imaging. Patients were split into three groups according to the pregnancy outcome: Biochemical Group (n=30 cycles and 54 transferred embryos), consisting of patients with a biochemical pregnancy; Positive Group (n=255 cycles and 444 transferred embryos), consisting of patients with a positive pregnancy result (clinical pregnancy); and Negative Group (n=468 cycles and 750 transferred embryos), consisting of patients with a negative pregnancy result (Figure 1).

All patients signed a written informed consent form in which they agreed to share the outcomes of their cycles for research purposes, and the study was approved by the local Institutional Review Board.

### Inclusion criteria

To be eligible for inclusion in this study, each patient or cycle had to meet the following inclusion criteria: pre-menopausal women between 35 and 40 years of age, undergoing COS with r-FSH for ICSI. Infertile women diagnosed with tubal infertility or unexplained infertility, or with partners diagnosed with male infertility factors, eligible for ICSI using fresh sperm from partner ejaculation; regular menstrual cycles every 25-35 days; body mass index between 18.5-24.9, calculated according to the following formula: body weight (kg) / (height x height (m^2^); presence of both ovaries; no clinically significant illness or pelvic and / or uterine abnormality; normal cervical cytology; serum FSH within normal limits (≤ 10 mU / ml); the study cycle was the first cycle of COS for ICSI; use of GnRH antagonist to prevent premature LH surge; use of recombinant human Chorionic Gonadotropin (r-hCG) to induce final oocyte maturation; culture of all embryos to D5.

### Exclusion criteria

To be eligible for inclusion in this study, the patient may not present with any of the following exclusion criteria: known stage III-IV endometriosis; known history of recurrent pregnancy loss (defined as two consecutive losses after ultrasound confirmation of pregnancy, excluding ectopic pregnancy, and before week 24 of pregnancy); known abnormal karyotype of the subject or his partner; any known clinically significant systemic disease (e.g., insulin-dependent diabetes); known inherited or acquired thrombophilia; known active arterial or venous thromboembolism, severe thrombophlebitis or a history of these events; known porphyria; known tumors in the ovaries, breasts, uterus, adrenal gland, pituitary or hypothalamus that would make the use of gonadotropins contraindicated; known moderate or severe impairment of kidney or liver function; undiagnosed vaginal bleeding; current known pelvic inflammatory diseases.

### Controlled Ovarian Stimulation and Laboratory Procedures

On the third day of the cycle, controlled ovarian stimulation was started by the administration r-FSH (300IU follitropin alpha, Gonal-F, Serono, Geneva, Switzerland or 16 mcg follitropin delta, Rekovelle^**®**^, Ferring, Saint-Prex, Switzerland) daily doses. r-FSH dose was adjusted according to follicular development, which was monitored by ultrasound scan.

When at least one follicle ≥14 mm was visualized, pituitary blockage was performed using gonadotropin-releasing hormone (GnRH) antagonist (GnRHa, Cetrotide^**®**^; Merck KGaA, Darmstadt, Germany). When three or more follicles attained a mean diameter of ≥ 17 mm and adequate serum estradiol levels were observed, r-FSH and GnRH antagonist administrations were stopped, and final follicular maturation was triggered by the administration of recombinant human chorionic gonadotropin (r-hCG, 250 µg, Ovidrel^®^, Merck KGaA, Geneva, Switzerland). Oocyte retrieval was performed 37 hours later. Oocytes in metaphase II were selected for ICSI.

### Semen analysis and preparation

Semen samples were collected in the laboratory by masturbation and were prepared using a two-layered density gradient centrifugation technique (50% and 90% Isolate, Irvine Scientific, Santa Ana, CA, USA).

### Intracytoplasmic sperm injection

Intracytoplasmic sperm injection was performed according to Palermo *et al.* (1992). Sperm was selected at 400x magnification using an inverted Nikon Eclipse TE 300 microscope and injected into the oocytes in a micro-injection dish prepared with buffered medium (Global w/HEPES, LifeGlobal, Guilford, USA) covered with paraffin oil (Paraffin oil P.G., LifeGlobal), on an inverted microscope heated stage (37.0°C±0.5°C).

### Embryo culture

Injected oocytes were individually cultured in a 16 well culture dish (Embryoslide, Unisense Fertilitech, Aarhus, Denmark), in 360µl of continuous single-culture media (Global^®^ total^®^, LifeGlobal), overlaid with 1.8ml of mineral oil (Paraffin oil P.G., LifeGlobal) in a TL-monitored incubator (EmbryoScope+, Unisense Fertilitech, Aarhus, Denmark) set at 37◦C with an atmosphere of 6% O_2_ and 7.2% CO_2_ until day five of embryo development. The incubator high-definition camera was set up to record embryos’ images, in eleven focal planes, every 10 minutes. Recorded kinetic markers were timing to pronuclei appearance (tPNa) and fading (tPNf), timing to two (t2), three (t3), four (t4), five (t5), six (t6), seven (t7), and eight cells (t8), and timing to morulae (tM), to start blastulation (tSB) and to blastulation (tB). Durations of the second (cc2, t3-t2) and third cell cycles (cc3, t5-t3) and timing to complete synchronous divisions t2-tPNf (s1), t4-t3 (s2) and t8-t5 (s3) were calculated. Data generated from EmbryoScope+ was analyzed using the EmbryoViewer software (Vitrolife, Denmark).

### Clinical follow-up

Embryo transfer was performed on day 5 of embryo development and one or two embryos were transferred per patient, depending on embryo quality and maternal age.

### Clinical outcomes

A pregnancy test was performed 8 days post blastocyst transfer. Not pregnant was defined as a serum b-human chorionic gonadotropin (hCG) concentration of <5mIU/mL. Women with a positive pregnancy test, had a transvaginal ultrasound scan 2 weeks later. The clinical pregnancy was diagnosed upon detection of foetal heartbeat. A biochemical pregnancy loss was defined as a positive b-hCG concentration that declined spontaneously. The pregnancy rate was calculated per embryo transfer. The implantation rate is the number of gestational sacs with foetal heartbeats divided by the number of transferred embryos. Miscarriage was defined as clinical pregnancy loss before 20 weeks.

### Data analysis and statistics

The primary outcome measure was tB since it is the most advanced key-stage of embryonic development recorded in our center. Post hoc power analysis was calculated, given α of 5%, sample size of 1248 embryos that reached blastocyst stage at day 5 of development, and effect size for tB. The achieved power was superior to 80%. The calculation was performed using Hotelling Lawley Trace test in GLIMMPSE App for multilevel data (15), which accounted for correlation between embryos from the same cycle.

Generalized mixed models (GMM), adjusted for potential confounders, were used to compare embryos morphokinetic behavior among the groups. Maternal and paternal ages were included as covariates in all models to control for their influence.

A random effect was added to account for the correlation between the embryos within the same cycle, with linear distribution for morphokinetic data in hours (h) and known implantation diagnosis score (KIDScore) ranking.

The results are expressed as the mean±standard error (SD), beta coefficient (B) with 95% confidence interval (CI), and p-values. *p*<0.05 was considered statistically significant. Data analysis was conducted using the Statistical Package for the Social Sciences (SPSS) 21 (IBM, New York, NY, USA).

## RESULTS

Comparisons of demographic data and ICSI cycles’ characteristics between the three groups are given in Table 1. Apart from maternal age that was significantly different between the Negative and Positive groups (38.0±0.1y-old *vs*. 37.2±0.2y-old, *p*=0.016, respectively) and paternal age that significantly differed between the Biochemical and the Negative and Positive groups (43.2±1.3y-old *vs*. 38.5±0.4y-old *vs*. 38.6±0.5y-old, *p*=0.003, respectively), the characteristics were similar among the groups.

**Table 1:** Comparison of demographic data and ICSI cycles’ characteristics between Biochemical, Positive and Negative groups (n=753 cycles).

ICSI outcomes	Biochemical Group	Negative Group	Positive Group	*p*-value
n	30	468	255	
Maternal age (years)	38.2±0.6 ^ab^	38.0±0.15 ^a^	37.2±0.2 ^b^	0.016
Paternal age (years)	43.2±1.3 ^a^	38.5±0.4 ^b^	38.6±0.5 ^b^	0.003
Female BMI (kg/m^2^)	23.6±1.4	24.2±0.4	24.2±0.5	0.916
Total dose of FSH (IU)	2200.0±349.9	2375.4±92.8	2455.5±121.2	0.739
Aspirated follicles (n)	8.0±1.4	8.1±0.4	9.8±0.5	0.687
Retrieved oocytes (n)	7.0±1.1	6.9±0.3	7.5±0.4	0.412
Mature oocytes rate (%)	77.4	76.7	74.3	0.795
Fertilization rate (%)	86.7	76.1	78.4	0.176
Blastocyst development (%)	43.0	41.0	44.7	0.599
Transferred embryos (n)	1.8	1.7	1.7	0.696

Note: ICSI-intracytoplasmic sperm injection; BMI-body mass index; FSH-follicle-stimulating hormone; IU-international unit. Values are the mean±standard error, unless otherwise noted. Different superscript letters in a line represent significant differences (*p*<0.05).

Comparisons of embryo morphokinetic parameters between the three groups are given in Table 2. The results from the generalized mixed models followed by Bonferroni post hoc for the comparison of means among groups showed that embryos resulting in biochemical pregnancy behaved similarly to those embryos resulting in a clinical pregnancy in terms of tPNa, tPNf, t2, t3, t4, t5, t6, t7, t8, tM, tsB, tB, cc2, cc3, s1, s2 and s3 and KIDScore ranking.

**Table 2:** Comparison of embryo morphokinetic parameters between Biochemical, Positive and Negative groups (n=1248 embryos).

Morphokinetics	*Biochemical Group*	*Negative Group*	Positive group^*^	*p*-value
n	54	750	444	
**tPNa**	5.53±0.65 ^a^ B: -0.865 (CI: -2.227-0.496)	7.54±0.18 ^b^ B: 1.144 (CI: 0.562 -1.726)	6.40±0.23 ^a^	**<0.001**
**tPNf**	23.47±0.81 ^a^ B: -0.597 (CI: -2.284-1.089)	25.60±0.22 ^b^ B: 1.528 (CI: 0.824-2.232)	24.07±0.28 ^a^	**<0.001**
**t2**	25.99±0.85 ^a^ B: -0.584 (CI: -2.352-1.184)	28.38±0.23 ^b^ B: 1.805 (CI: 1.067-2.542)	26.58±0.30 ^a^	**<0.001**
**t3**	36.70±0.93 ^a^ B: -1.356 (CI: -3.283-0.571)	39.00±0.25 ^b^ B: 0.953 (CI: 0.149-1.758)	38.05±0.32 ^a^	**0.008**
**t4**	39.00±1.03 ^a^ B: -0.061 (CI: -2.201-2.079)	41.57±0.28 ^b^ B: 2.514 (CI: 1.621-3.407)	39.06±0.36 ^a^	**<0.001**
**t5**	50.46±1.21 B: -0.015 (CI: -2.214-2.484)	51.59±0.32 B: 1.120 (CI: 0.097-2.142)	50.47±0.41	0.085
**t6**	52.68±1.34 ^ab^ B: -0.100 (CI: -2.880-2.680)	54.87±0.36 ^b^ B: 2.098 (CI: 0.950-3.246)	52.78±0.46 ^a^	**0.001**
**t7**	55.53±1.30 ^ab^ B: 0.412 (CI: -2.282-3.106)	57.45±0.36 ^b^ B: 2.327 (CI: 1.209-3.445)	55.12±0.44 ^a^	**<0.001**
**t8**	58.58±1.44 ^ab^ B: 0.839 (CI: -2.149-3.828)	61.28±0.40 ^b^ B: 3.550 (CI: 2.294-4.806)	57.74±0.50 ^a^	**<0.001**
**tM**	87.78±1.54 B: 0.323 (CI: -2.860-3.505)	88.20±0.44 B: 0.739 (CI: -0.587-2.065)	87.46±0.51	0.549
**tsB**	99.03±1.29 B: 1.363 (CI: -1.306-4.032)	98.58±0.38 B: 0.917 (CI: -0.229-2.064)	97.67±0.44	0.239
**tB**	107.93±1.23 B: 2.722 (CI: 0.173-5.271)	105.97±0.39 B: 0.765 (CI: -0.386-1.915)	105.20±0.43	0.080
**cc2**	10.63±0.14 ^a^ B: -0.842 (CI: -1.279--0.405)	11.47±0.18 ^b^ B: 1.172 (CI: 0.811-2.275)	10.70±0.50 ^a^	**0.001**
**cc3**	13.32±0.75 B: 0.879 (CI: -0.680-2.438)	12.73±0.20 B: 0.287 (CI: -0.351-0.925)	12.44±0.26	0.448
**s1**	2.52±0.12 ^ab^ B: 0.014 (CI: -0.238-0.266)	2.66±0.03 ^b^ B: 0.156 (CI: 0.051-0.261)	2.51±0.04 ^a^	**0.012**
**s2**	2.30±0.56 ^ab^ B: 1.295 (CI: 0.139-2.451)	2.57±0.15 ^b^ B: 1.561 (CI: 1.078-2.043)	1.01±0.19 ^a^	**<0.001**
**s3**	8.12±1.16 ^ab^ B: 0.654 (CI: -1.752-3.060)	10.55±0.33 ^b^ B: 3.090 (CI: 2.079-4.101)	7.46±0.40 ^a^	**<0.001**
**KIDScore**	5.59±0.35 ^a^ B: -0.397 (CI: -1.133-0.340)	4.73±0.11 ^b^ B: -1.257 (CI: -1.583--0.932)	5.99±0.13 ^a^	**<0.001**

Note: tPNa-timing to pronuclei appearance, tPNf-timing to pronuclei fading, t2-timing to two cells, t3-timing to three cells, t4-timing to four cells, t5-timing to five cells, t6-timing to six cells, t7-timing to seven cells, t8-timing to eight cells, tSB-timing to start blastulation, tB-timing to blastulation, s1-timing to complete t2-tPNf synchronous divisions, s2-timing to complete t4-t3 synchronous divisions, s3-timing to complete t8-t5 synchronous divisions, cc2-duration of the second cell cycle (t3-t2), cc3-duration of third cell cycle (t5-t3), KIDScore-known implantation diagnosis score. Values are the mean±standard error, unless otherwise noted. Different superscript letters in a line represent significant differences (*p*<0.05). B-regression coefficient, CI-95% confidence interval.

* Reference group.

Embryos resulting in a negative pregnancy showed significantly slower embryo development in terms of tPNa, tPNf, t2, t3, t4, cc2, and KIDScore ranking compared to both Biochemical and Positive groups and differed from the Positive group only in terms of t6, t7, t8, s1, s2, and s3.

## DISCUSSION

Biochemical pregnancy may occur due to potential disruptions in the intricate communication between the embryo and uterus during implantation, which could lead to reduced chances of successful reproduction. However, the factors associated with BP following ART have not been thoroughly investigated. From the onset of implantation, a natural selection process is in place that prevents embryos with low viability from advancing past the peri-implantation stage. Not only do decidualized stromal cells in the endometrium play a wide role in vascular restructuring and immune response regulation, but they also serve as detectors for signals originating from the embryo prior to implantation. In the present study we hypothesized that the TLI technology, which has allowed the possibility of assessing complete embryonic development, could be used to identify potential morphokinetic characteristics of a developing embryo associated with the occurrence of a BP. Our results showed that embryos that resulted in a BP did not display evidence of abnormal morphokinetics, as they behaved more similarly to those embryos resulting in a clinical pregnancy than to those resulting in a negative outcome.

In fact, it has been demonstrated that the occurrence of biochemical pregnancy is linked to elements that modify the implantation process at the endometrial level, rather than solely the embryo’s chromosomal status. Given that the standard implantation process involves a sequence of interactions between the embryo and the endometrium, changes in endometrial receptivity could be the primary reason for biochemical pregnancy in ART cycles (Troncoso *et al.*, 2003). In a previous study performed by our group, the incidence of biochemical pregnancy in ICSI cycles was associated with poor endometrial receptivity, supraphysiological hormone levels and poor seminal parameters (Zanetti *et al.*, 2019).

On the other hand, in the present study we observed a slower morphokinetic development in embryos that failed to implant, when compared to embryos that resulted in biochemical and clinical pregnancy, which was confirmed by significant differences found in KIDScore ranking among the groups. The Negative group took significantly longer to reach several milestones as early as tPNa. Indeed, it seems that the initial embryonic cleavages were particularly slower in the negative group. It has been demonstrated that faster tPNf and t2 were associated with better embryo morphology on day 3 (Coticchio *et al.*, 2018), shorter t4 was specifically correlated with euploidy (Minasi *et al.*, 2016), and embryos that cleave faster from the 2- to 8-cell stage have a higher potential for blastulation, implantation and live-birth compared to those that divide more slowly (Dal Canto *et al.*, 2012; 2021; Kirkegaard *et al.*, 2013; Meseguer *et al.*, 2011).

This study design faces several key limitations that could affect its validity and generalizability. The retrospective nature of the study and the small sample size are the main limitations of this study, which may limit causal inferences and reduce the statistical power and generalizability of results, respectively. The unequal group sizes, with a particularly small biochemical pregnancy group, limit statistical power and may skew comparisons. Despite the eligibility criteria for inclusion in the analysis, potential differences in the baseline characteristics cannot be ruled out, and the lack of control for confounding factors such as sperm quality reduces the reliability of observed associations, and the sole focus on pregnancy outcomes without assessing live birth rates diminishes the clinical relevance. Furthermore, conducting the study in a private IVF center restricts its external validity, as the patient population may not represent broader demographics. The reliance on a single TLI incubator system (EmbryoScope+) raises questions about the generalizability of findings to other incubators. Finally, failing to account for potential temporal biases from the multi-year study period could confound results if IVF protocols or technologies evolved over time. Therefore, a cautious interpretation is due.

On the other hand, this study has several strengths that enhance its robustness and relevance in investigating embryo development kinetics. The use of a large dataset, including 1,248 transferred embryos from 753 patients, provides substantial statistical power to detect meaningful patterns and associations. By adjusting for clustering due to pooling of embryos from the same patient, the analysis accounts for the non-independence of data points, ensuring more accurate and reliable results. The study’s reliance on TLI using a standardized incubator (EmbryoScope+) allows for precise, consistent monitoring of embryo development, minimizing variability due to observational bias. Additionally, the classification of patients into three clinically relevant outcome groups (biochemical, positive, and negative pregnancies) facilitates nuanced insights into how kinetic parameters might differ across varying outcomes. Conducting the research over a three-year period also strengthens the findings by incorporating a broad dataset reflective of real-world IVF practices over time. These strengths position the study as a valuable contribution to understanding how embryonic kinetics relate to pregnancy outcomes in a controlled clinical setting.

This study provides valuable insights into the relationship between embryonic development kinetics and pregnancy outcomes. The finding that embryos resulting in BP exhibit developmental patterns and KIDScore rankings like those of embryos leading to clinical pregnancies emphasizes the complexity of BP, suggesting that it may not solely result from poor embryonic quality but could also involve multifactorial influences, including endometrial receptivity. The observed slower development and lower KIDScore rankings in embryos associated with negative pregnancy outcomes highlight the utility of TLI and embryo scoring systems in identifying embryos with limited implantation potential. By adjusting for clustering and analyzing a large, well-characterized dataset, this study not only underscores the potential of TLI for refining embryo selection but also raises critical questions about the role of maternal and environmental factors in BP. These findings pave the way for future research aimed at improving embryo selection protocols and developing strategies to predict and prevent biochemical pregnancies, ultimately advancing the success and personalization of assisted reproductive technologies.

In conclusion, embryos resulting in BP behaved similarly to those embryos resulting in a clinical pregnancy, while embryos resulting in a negative pregnancy showed significantly slower embryo development and KIDScore ranking compared to both Biochemical and Positive groups. Further research is needed to identify factors that can predict and prevent biochemical pregnancy.
